# Interactions between Nef and AIP1 proliferate multivesicular bodies and facilitate egress of HIV-1

**DOI:** 10.1186/1742-4690-3-33

**Published:** 2006-06-09

**Authors:** Luciana J Costa, Nan Chen, Adriana Lopes, Renato S Aguiar, Amilcar Tanuri, Ana Plemenitas, B Matija Peterlin

**Affiliations:** 1 Molecular Virology Laboratory, Dep. of Genetics, Federal University of Rio de Janeiro, Rio de Janeiro, Brazil; 2 Departments of Medicine, Microbiology and Immunology, Rosalind Russell Medical Research Center, University of California at San Francisco, San Francisco, CA, USA; 3 Institute of Biochemistry, Faculty of Medicine, University of Ljubljana, Ljubljana, Slovenia

## Abstract

**Background:**

Nef is an accessory protein of primate lentiviruses, HIV-1, HIV-2 and SIV. Besides removing CD4 and MHC class I from the surface and activating cellular signaling cascades, Nef also binds GagPol during late stages of the viral replicative cycle. In this report, we investigated further the ability of Nef to facilitate the replication of HIV-1.

**Results:**

To this end, first the release of new viral particles was much lower in the absence of Nef in a T cell line. Since the same results were obtained in the absence of the viral envelope using pseudo-typed viruses, this phenomenon was independent of CD4 and enhanced infectivity. Next, we found that Nef not only possesses a consensus motif for but also binds AIP1 *in vitro *and *in vivo*. AIP1 is the critical intermediate in the formation of multivesicular bodies (MVBs), which play an important role in the budding and release of viruses from infected cells. Indeed, Nef proliferated MVBs in cells, but only when its AIP1-binding site was intact. Finally, these functions of Nef were reproduced in primary macrophages, where the wild type but not mutant Nef proteins led to increased release of new viral particles from infected cells.

**Conclusion:**

We conclude that by binding GagPol and AIP1, Nef not only proliferates MVBs but also contributes to the egress of viral particles from infected cells.

## Background

Primate lentiviruses HIV-1, HIV-2 and SIV infect macrophages and T lymphocytes via CD4 and CCR5 or CXCR4 chemokine receptors, respectively. Infected individuals eventually develop the acquired immunodeficiency syndrome (AIDS). The course of their disease varies greatly, which depends on genetic factors and host immune responses [1,2]. Another important determinant of disease progression is the viral accessory protein, the misnamed negative factor or Nef. Indeed, adult rhesus macaques and humans infected with lentiviruses lacking Nef have very low levels of viral replication and little, if any, evidence of disease [3-5]. Only with the reconstitution of their nef genes do these viruses start to replicate robustly, which then leads to AIDS [6-8]. Thus, Nef has been considered a critical factor for the production and infectivity of primate lentiviruses in the host, which is a phenotype that is reproduced best in studies using primary cells in culture [9-12].

Nef is a small, myristylated protein that is expressed early in the viral replicative cycle. It is found on cellular membranes as a homodimer, where each subunit measures 27 to 32 kDa. Among all Nef proteins, the most conserved region is the central core domain of 6 α helices and 5 β sheets that binds many lipid, serine/threonine and tyrosine kinases as well as guanine nucleotide exchange factors and small GTPases [13]. The signalosome that is assembled on Nef leads to downstream effector functions and cytoskeletal rearrangements [14]. Near its N-terminus is the binding site for CD4 and the C-terminal flexible loop interacts with several subunits of adaptor protein (AP) complexes as well as with other trafficking molecules [15-20]. Thus, Nef also affects the movement of intracellular organelles. Of interest, these functions can be linked, as phosphoinositol 3-kinase (PI3K) also contributes to the sequestration of major histocompatibility complex (MHC) class I determinants [21].

In addition, Nef can accumulate in detergent resistant microdomains (DRMs) or lipid rafts [22], and is incorporated into new viral particles [23,24]. It also augments the infectivity of progeny virions, in part, by increasing the incorporation of lipids into viral membranes [25]. To this end, Nef not only induces the synthesis of cholesterol but carries this lipid into viral particles [25]. These viral particles then fuse with DRMs on the recipient cell [26]. To accomplish some of these chaperone functions, Nef binds the transframe p6* protein from GagPol, which does not exist in Gag [27]. Of interest, if Nef is retained near the endoplasmic reticulum (ER) either as a naturally occurring dominant negative Nef protein (NefF12) or by adding the ER-retention signal (KKXX) to Nef (NefKKXX), no viral particles are made and no Gag processing is observed [27,28]. Thus, by biochemical and genetic criteria, Nef binds GagPol and affects the replication of HIV-1 via its association with viral assembly intermediates.

Recently, Nef has been demonstrated to proliferate multivesicular bodies (MVBs) [29,30] and to facilitate the egress of a variety of pseudotyped viruses from cells [31]. These studies suggest that Nef contributes directly to the replication of HIV-1, possibly as a "modified" late (L) domain. L domains of retroviruses and other RNA viruses bind the tumor suppressor gene 101 (Tsg101) from the Endosomal Sorting Complex Required for Transport I (ESCRTI) [32-35] or the apoptosis linked gene 2 (ALG2)-interacting protein 1 (AIP1) that bridges ESCRTI and ESCRTIII [36-39]. With the help of PI3K, phosphoinositol 3 phosphate (PI3P), AAA ATPase Vps4, these E-Vps or ESCRT proteins then create vacuoles into which vesicles bud [40-42]. Indeed, these interactions are required for the successful morphogenesis and release of viruses from infected cells. In the case of HIV-1, whereas p6 from Gag binds both Tsg101 and AIP1, p6* from GagPol contains a completely different sequence and no such consensus binding motif. However, we found that its binding partner, Nef, not only contains such a site and binds AIP1 but that it proliferates MVBs and leads to increased production of viral particles from transformed cell lines and primary macrophages. Thus, Nef can contribute directly to the egress of HIV-1 from infected cells.

## Results

### Nef increases levels of HIV-1 produced from SupT1 cells by a mechanism that is independent of CD4 and enhancement of viral infectivity

Previously, we demonstrated that Nef binds GagPol from HIV-1 during late stages of the viral replicative cycle [27]. To determine what role this binding plays for the virus, several CD4-positive cells were examined for the replication of HIV-1 in the presence and absence of Nef. Initially, SupT1, Jurkat, CEM and MOLT4 cells were electroporated with plasmids that directed the expression of HIV-1_NL4-3 _and mutant HIV-1_NL4-3_ΔNef proviruses and virus production was measured 2 to 8 days later, both by levels of p24 capture ELISA and by western blotting of purified viruses with α p24 antibodies. At day 2, we observed an 8-fold decreased release of viral particles from SupT1 cells transfected with the mutant

**Figure 1:**
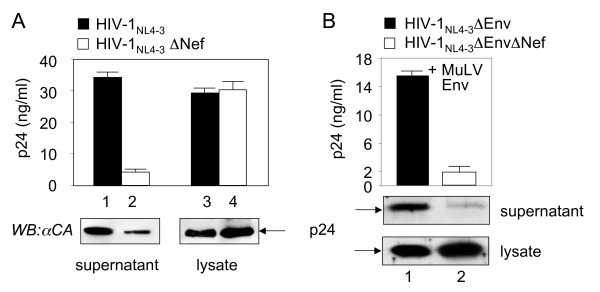
**Nef increases levels of HIV-1 produced from SupT1 cells by a CD4 independent mechanism**.**A) **SupT1 cells (1 × 10^7^cells) were electroporated with 10 μg of plasmids directing the expression of wild type HIV-1_NL4-3 _and mutant HIV-1_NL4-3_ΔNef proviruses. 2 days later, supernatants and cells were collected and p24 levels were measured by p24 capture ELISA (top panel). Viruses from cell supernatants were concentrated by ultracentrifugation. Viruses and cell lysates were processed for western blotting (WB) with α p24 antibodies (bottom panel). Bar graphs contain: Black bars, wild type HIV-1_NL4-3 _provirus; white bars, mutant HIV-1_NL4-3_ΔNef provirus. Errors bars denote differences between three experiments performed in duplicate. **(B) **SupT1 cells (1 × 10^7^cells) were electroporated with 10 μg of plasmids directing the expression of mutant HIV-1_NL4-3_ΔEnv and HIV-1_NL4-3_ΔEnvΔNef proviruses together with 5 μg of an expression plasmid for the MuLV Env glycoprotein (MuLV Env). 2 days later, supernatants and cells were collected and p24 levels were measured as in (A). Error bars are as in (A).

HIV-1_NL4-3_ΔNef provirus when compared to its wild type HIV-1_NL4-3 _counterpart, whereas intracellular viral production was at the same levels for both proviruses (Fig. 1A, compare lanes 1 to 4). The earlier time point is presented because at 2 days, we observed only a single round of viral replication. Of interest, this decreased egress of mutant HIV-1_NL4-3_ΔNef viral particles was not observed in Jurkat, CEM and MOLT4 cells (data not presented). These findings are in agreement with previous studies demonstrating the importance of Nef for the production of HIV-1 from SupT1 cells [43,44].

Since it was reported that Nef facilitates the release of HIV-1 in T cells by decreasing the expression of CD4 on the cell surface [45,46], a possible explanation for our finding would be that SupT1 cells contain higher amounts of CD4. In these studies, by binding HIV-1 Env, CD4 blocked the release of new viral particles and/or prevented the infection of new cells via CD4 [45,46]. To exclude this possibility, we pseudotyped mutant HIV-1_NL4-3_ΔEnv and

HIV-1_NL4-3_ΔEnvΔNef proviruses that lack HIV-1 Env with Env from the murine leukemia virus (MuLV Env) that does not bind CD4, and obtained identical results (Fig. 1B). Again, at day 2 after the transfection, levels of p24 in the supernatant from these SupT1 cells were 8-fold higher in the presence than in the absence of Nef (Fig. 1B, compare lanes 1 and 2). Importantly, the MuLV Env does not support a second round of viral replication in SupT1 cells. Identical results were obtained when no Env was co-expressed with HIV-1_NL4-3_ΔEnv and HIV-1_NL4-3_ΔEnvΔNef proviruses (data not provided). Thus, these assays do not measure effects of Nef on the infectivity of HIV-1. This result confirms that Nef is required for the egress of HIV-1 by a mechanism other than the removal of CD4 from HIV-1 Env and emphasizes the importance of Nef during late stages of the viral replicative cycle in these cells.

### Nef can substitute for the function of the L domain of Gag

**Figure 2:**
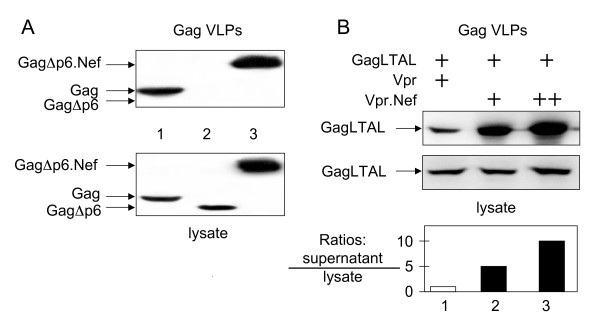
**Nef rescues the release of Gag VLPs from the L domain-deleted and L domain-mutated Gag polyproteins**.**A)Efficient production of Gag VLPs from a mutant hybrid GagΔ p6.Nef chimera. **Two days after the transfection, supernatants from 293T cells expressing wild-type Gag as well as mutant GagΔ p6 proteins and the mutant hybrid GagΔ p6.Nef chimera were collected and submitted to ultracentrifugation for the purification of Gag VLPs. Purified Gag VLPs and cell lysates were processed as in Fig. 1. Lane 1: Wild type Gag protein; Lane 2: Mutant GagΔ p6 protein; Lane 3: Mutant hybrid GagΔ p6.Nef chimera. **(B)Hybrid Vpr.Nef protein increases the release of Gag VLPs from a mutated p6 and Pol-deleted virus. **The mutant GagLTAL provirus was co-expressed with Vpr or with the Vpr.Nef chimera in 293T cells. Two days after the transfection, supernatants and cells were collected. Purified Gag VLPs and cell lysates were processed as in Fig. 1. Equivalent amounts of the mutant GagLTAL protein were loaded in the lysate to facilitate comparisons between GagVLPs in the supernatant. Gag VLPs were detected with α p24 antibodies. Ratios between the mutant GagLTAL proteins in supernatants and lysates are presented in the bar graph below the western blots. Lane 1: Mutant GagLTAL protein with Vpr; Lanes 2 and 3; Mutant GagLTAL protein and increasing concentrations of the hybrid Vpr.Nef protein.

The budding of HIV-1 is dependent on the consensus Tsg101-binding motif (PTAP), which is located in p6 of Gag [33]. To confirm that Nef could contribute to the release of viral particles, we examined the ability of Nef to rescue the production of VLPs from mutant Gag proteins (Gag VLPs) with deletions (GagΔ p6) or mutations (GagLTAL) in the L domain. As presented in Fig. 2A, very low levels of Gag VLPs were detected in supernatants from cells, which expressed GagΔ p6 alone (lane 2). However, when Nef was linked to the C-terminus of the mutant GagΔ p6 polyprotein (GagΔ p6.Nef), the production of Gag VLPs was restored to wild type levels (Fig 2A, compare lanes 1, 2 and 3). Intracellular levels of wild type Gag, mutant GagΔ p6 and mutant hybrid GagΔ p6Nef proteins are presented in the bottom panel of Fig. 2A. Thus, Nef can substitute for the function of the L domain for the production of Gag VLPs.

For the second strategy, Nef was expressed as a hybrid Vpr.Nef protein, because the binding site for Vpr within Gag is preserved in the mutant GagLTAL protein. Thus, Vpr should bring Nef to Gag. When the mutant GagLTAL protein was expressed with Vpr, a very inefficient production of Gag VLPs was observed from 293T cells (Fig. 2B, lane 1). However, the co-expression of the mutant GagLTAL protein with increasing amounts of the Vpr.Nef chimera augmented the release of these Gag VLPs (Fig 2B, top panel, compare lanes 1, 2 and 3). We loaded equivalent amounts of the mutant GagLTAL protein in the lysate so that increased levels of Gag VLPs in the supernatant could be compared directly (Fig. 2B, top and bottom panels, compare lanes 1, 2 and 3). For the graph at the bottom of Fig. 2B, which presents ratios between mutant GagLTAL proteins in supernatants and lysates, amounts of mutant GagLTAL proteins were measured by densitometry of different exposures of these western blots. From this graph (Fig. 2B, bottom), we conclude that the Vpr.Nef chimera can increase the release of these Gag VLPs up to 10-fold. Thus, Nef can promote the egress of HIV-1 and Gag VLPs from cells.

### Nef contains a consensus-binding site for AIP1

**Figure 3:**
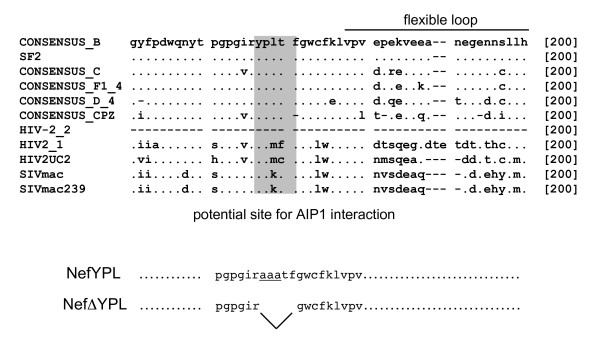
**Nef contains the consensus-binding site for AIP1**. Multiple alignments of sequences were generated by the Clustal W software and visually inspected for the presence of already described L domain motifs [47]. The AIP1-consensus binding site is highlighted. Consensus residues represent several subtypes of HIV-1. Below them are Nef sequences from HIV-2 and SIV that do not contain this consensus sequence. AIP1 binds elsewhere on these proteins. These sequences are from the Los Alamos database [48]. Below these sequences are diagrammed mutations that were introduced into Nef, one mutating the YPL sequence to three alanines (NefYPL), the other deleting the entire consensus motif (NefΔ YPL).

From these results, we hypothesized that Nef could function as a "modified" L domain by helping to connect viral assembly intermediates to the components of the ESCRT machinery involved in HIV-1 budding. To confirm this hypothesis we first generated multiple alignments of Nef using the Clustal W algorithm [47,48] and inspected them visually for the presence of sequences resembling the already described L domain-binding motifs. We found the YPLT sequence (residues from positions 135 to 138), close to the C-terminal flexible-loop of Nef (Fig. 3). This sequence resembles the YPLTS domain described as an AIP1-binding site in p6 from HIV-1 and p9 from EIAV [36]. It is important to note that this sequence has a high degree of conservation among all isolates of HIV-1 but not of HIV-2 and SIV (Fig. 3). Rather, Nef proteins from these related lentiviruses contain another consensus AIP1-binding site at their N-termini (data not presented), which has been implicated recently in high levels of SIV replication in rhesus macaques [49].

### Nef binds AIP1 in vitro and in vivo

**Figure 4:**
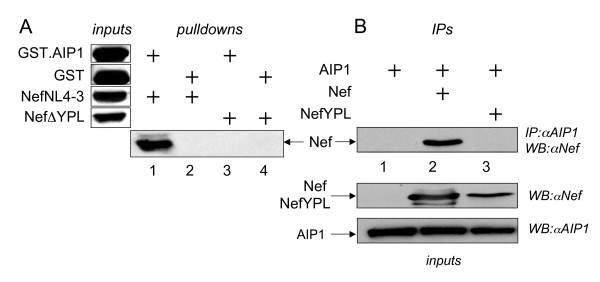
**Nef binds AIP1 *in vitro *and *invivo***.**(A)Nef binds AIP1 *in vitro*. **GST and GST.AIP1 fusion proteins were expressed in *E. coli *and purified by glutathione S-transferase beads. They were incubated with V5 epitope-tagged wild type Nef and mutant NefΔ YPL proteins expressed in IVT. Bound proteins were resolved by 10% SDS-PAGE followed by western blotting with α V5 antibodies. GST was used as the negative control (top right panel, lane 2). 10% of input proteins (inputs) is presented to the left of GST pulldowns. **(B) Nef binds AIP1 in cells. **HA epitope-tagged AIP1 protein was expressed alone or with the wild type and mutant NefYPL proteins in 293T cells. Cells were disrupted by dounce homogenization in hypotonic buffer containing protease inhibitor cocktails, followed by incubation with α HA polyclonal antibodies and protein-G beads. After the immunoprecipitation, western blotting was performed using α Nef antibodies (top left panel). A control western blot for 10% of input proteins was performed with α Nef and α AIP1 antibodies (bottom left panels).

Next, we investigated the ability of Nef to bind AIP1. To detect this binding, plasmids directing the expression of wild type and mutant Nef proteins at the putative consensus AIP1-binding site were generated. Whereas the mutant NefΔ YPL protein contains a deletion of this motif, in the mutant NefYPL protein, the YPL sequence has been replaced by three alanines (Fig. 3, bottom). All Nef proteins were expressed from the coupled transcription and translation reactions with rabbit reticulocyte lysates in vitro (IVT) (Fig. 4A, inputs). AIP1 was expressed and purified as the GST.AIP1 chimera from *E. coli*. GST alone was expressed likewise and used as the negative control (Fig. 4A, inputs). Subsequent GST pulldowns revealed that Nef binds AIP1 (Fig. 4A, lanes 1 and 2). Since the deletion of the YPLTF sequence in the mutant NefΔ YPL protein abolished this binding, this interaction was also specific (Fig. 4A, lanes 3 and 4). Thus, Nef binds AIP1 and its consensus AIP1-binding site is required for this interaction in vitro.

This binding was confirmed by co-immunoprecipitations in cells. 293T cells co-expressed AIP1 and Nef proteins, which were immunoprecipitated with α AIP1 antibodies. After SDS-PAGE and transfer to membranes, western blotting with α Nef antibodies revealed Nef-specific bands (Fig. 4B). Again, AIP1 was only able to precipitate the wild type but not mutant NefYPL proteins (Fig. 4B, compare lanes 1, 2 and 3). Importantly, wild type and mutant Nef proteins were expressed robustly in cells. Additionally, since their migration patterns did not change, these mutations most likely do not affect the structure of the protein. Of note, similar confirmatory deletions and mutations were used to map the AIP1-binding site in p6 [36]. Importantly, two independent approaches with two complementary mutant Nef proteins yielded identical results. We conclude that Nef from HIV-1 binds AIP1 specifically in vitro and in vivo.

### Interactions between Nef and AIP1 are required for the proliferation of MVBs

**Figure 5:**
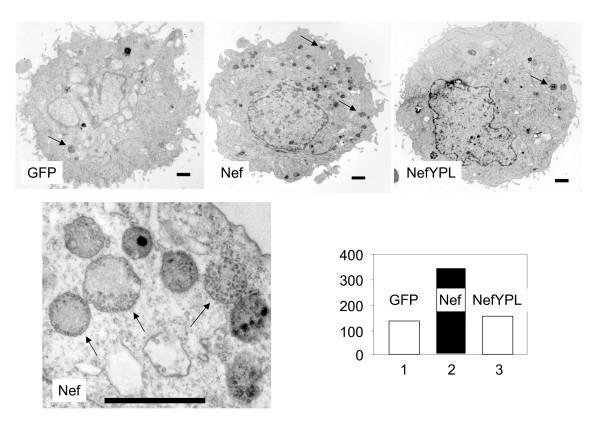
**Interactions between Nef and AIP1 are required for the proliferation of MVBs**. HeLa.CIITA cells were transfected with plasmids, which directed the expression of GFP, Nef.GFP, or mutant NefYPL.GFP chimeras (top panels). GFP-positive cells were isolated by FACS and fixed before ultra-thin sectioning was performed. MVBs were identified by their unique morphology (bottom left panel) under the electron microscope (indicated by arrows). Numbers of MVBs of each cell type were counted directly under the electron microscope from 30 profiles randomly taken from each sample. Bar graphs contain: White bars, GFP control; black bars, Nef; striped bars, mutant Nef.YPL protein.The black bar inside the EM panels measures 1 μm.

It had been demonstrated that Nef increases the accumulation of late endosomes in CEM and SupT1 cells [30]. More recently, Nef induced the proliferation of MVBs in HeLa.CIITA cells [29]. Given that AIP1 plays an important role in the formation of MVBs, we investigated if this finding results from interactions between Nef and AIP1. Thus, we expressed GFP, wild type Nef.GFP and mutant NefYPL.GFP chimeras in HeLa.CIITA cells. Cell expressing GFP were isolated by FACS, fixed and processed for electron microscopy. Under the electron microscope, MVBs can be identified by their unique morphological appearance, higher electron density and tightly packed internal vesicles, which distinguishes them from other organelles (Fig. 5A, bottom left panel) [29]. The number of MVBs in each cell was counted directly under the electron microscope from 30 images taken randomly from each sample. Thus, at least 30 cells were examined and findings from three independent experiments were averaged (Fig. 5A, bottom right panel). In agreement with the previous publication [29], the expression of the wild type Nef protein increased the accumulation of MVBs 3-fold in HeLa.CIITA cells (Fig. 5, top and right bottom panels). Remarkably, this effect was abolished with the mutant NefYPL protein, which no longer binds AIP1. Indeed, in cells expressing the mutant NefYPL.GFP chimera, the number of MVBs was similar to that in control cells that expressed only GFP. Thus, the proliferation of MVBs requires interactions between Nef and AIP1.

### Interactions between Nef and AIP1 are required for increased production of HIV-1 by Nef in primary macrophages

**Figure 6:**
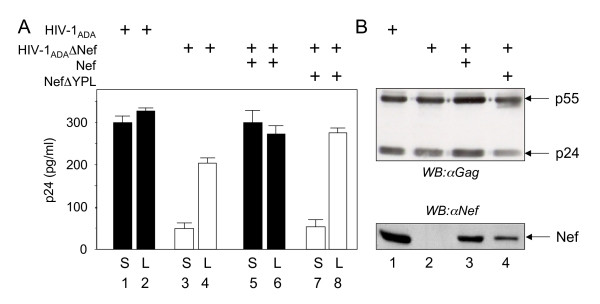
**Interactions between Nef and AIP1 increase the production of HIV-1 from primary macrophages**. **(A) Only the wild type Nef protein can rescue the production of mutant viruses in macrophages. **Macrophages were derived from PBMCs by adherence to plastic in the presence of 5% human serum. 7 days after differentiation, macrophages were transfected with wild type HIV-1_ADA _and mutant HIV-1_ADA_Δ Nef proviruses, or co-transfected with HIV-1_ADA_Δ Nef provirus with the wild type Nef or mutant NefΔ YPL proteins. 5 days after the transfection, supernatants (S) and cell lysates (L) were examined for the presence of viral particles by the p24 capture ELISA. Bar graphs contain: Black bars, HIV-1_ADA _alone or the mutant HIV-1_ADA_Δ Nef provirus with Nef; white bars, the mutant HIV-1_ADA_Δ Nef provirus; striped bars, the mutant HIV-1_ADA_Δ Nef provirus with the mutant NefΔ YPL protein. Errors bars denote differences between 5 independent experiments performed with the CaPO4 transfection protocol. **(B)Expression of wild type and mutant viruses and wild type and mutant Nef proteins were equivalent in cells.**Cell lysates from transfected macrophages were obtained concurrently and processed as in Figs. 1, 2, and 4.

Mature viral particles accumulate inside late endosomes in human mononuclear cells [50]. Later, the site of HIV-1 budding was proved to be in MVBs in macrophages [29,51]. Since by binding AIP1, Nef proliferates MVBs, we investigated further viral replication in primary macrophages, which were derived from peripheral blood mononuclear cells (PBMCs). Macrophages were allowed to differentiate for 7 days. They were transfected and then harvested 5 days later. Similar to data in Fig. 1, we observed that in the absence of Nef, the production of the mutant R5 virus, HIV-1_ADA_Δ Nef, was up to 6-fold lower than of its wild type counterpart (HIV-1_ADA_) in primary macrophages (Fig. 6A, compare bars 3, 4, 7 and 8). Furthermore, the co-expression of the wild type but not mutant NefΔ YPL proteins with the mutant HIV-1_ADA_Δ Nef provirus rescued the production of progeny virions to the same levels as were observed with the wild type HIV-1_ADA _provirus (Fig. 6A, compare bars 1, 2, 5 and 6). These experiments were repeated a total of 5 times with identical results. Western blotting from cell lysates demonstrated that levels of Gag and Nef were matched in cells expressing the wild type and mutant HIV-1_ADA_Δ Nef proviruses (Fig. 6B, top and bottom panels), confirming that the block in viral production was at a later step. Although initial experiments were performed using lipofectamine to transfect primary macrophages, the resulting levels of p24 were low. Nevertheless, a total of 8 independent experiments with lipofectamine also demonstrated the same effects of Nef. Subsequently, these studies were repeated using CaPO_4_, which led to 5-fold better tranfection efficiencies (Fig. 6). Nevertheless, levels of expression remained somewhat lower in our transfected than have been observed in infected macrophages [51]. Identical results were obtained when we used another R5 virus, the wild type HIV-1_ELI _and mutant HIV-1_ELI_Δ Nef proviruses (data not presented). Thus, Nef also increases the production of HIV-1 from primary macrophages.

## Discussion

In this report, we studied effects of Nef on the proliferation of MVBs and increased production of HIV-1 from infected cells. Whereas in SupT1 cells and primary macrophages, Nef increased the extracellular accumulation of new viral particles, in 293T cells, Nef rescued the production of Gag VLPs from mutant GagΔ p6 or Gagp6LTAL proteins, which lacked the L domain. This phenotype was correlated with interactions between Nef and AIP1, which were documented by GST pulldowns and co-immunoprecipitations in cells. Importantly, this association was specific, as mutations in the conserved YPL motif in Nef abolished this binding and eliminated effects of Nef on the proliferation of MVBs and release of viral particles. We conclude that by connecting GagPol and AIP1, Nef acts as a chaperone the production and optimal egress of HIV-1 from infected cells.

Importantly, we used a transformed cell line as well as primary cells, especially since effects of Nef are most pronounced in PBMCs and in the infected host [3-12]. Since we did not observe the same phenotype in Jurkat, CEM and Molt4 cells, the targeting of viral assembly intermediates to the cell surface rather than intracellular organelles must also be more efficient in these cells. Indeed, in sharp contrast to macrophages, no budding into MVBs had been observed in these other T cell lines [50,51]. Importantly, a role for CD4 could be excluded since the egress of pseudotyped viral particles, which contained the MuLV Env that does not bind CD4 instead of HIV Env, from SupT1 cells and that of wild type progeny virions from macrophages that express low levels of CD4, were impacted identically by Nef. In addition, it was important to confirm this effect of Nef with mutant Gag proteins bearing deletions or mutations in p6, as this assay represents an important genetic proof for interactions between viral proteins and the ESCRT machinery [27,33]. We also confirmed the specificity of binding for AIP1 by deletions and mutations of the consensus YPL motif in Nef. For morphological studies, we used HeLa.CIITA cells, which express the class II transactivator (CIITA) and hence MHC class II [52]. There were several reasons for this choice. First, the effect of Nef on the proliferation of MVBs had been documented in these cells [29]. Second, they contain MHC class II compartments (MIICs), which are MVBs for antigen processing and presentation by this pathway. Since their composition had been examined extensively in these cells, we could conclude that our dense vacuoles filled with vesicles were MVBs by morphological criteria alone [29,53]. In addition, increased levels of MVBs in our study were identical to those already reported [29,30]. Importantly, the mutation of the AIP1- binding site in Nef abolished this proliferation.

How do these findings fit into our view of Nef? Although effects of Nef in infected cells are multifactorial, above all, Nef is required for high levels of viral replication and the progression to AIDS in the infected host [3-5]. In primary cells, Nef also increases levels and infectivity of progeny virions [12,54,55]. Cellular activation by Nef has been implicated in low but detectable levels of viral replication in unstimulated PBMCs [22,56]. However, even after the stimulation with PHA, levels of progeny virions from mutant HIV-1Δ Nef proviruses are still 5-fold lower when compared to those with wild type proviruses in PBMCs [57]. These findings suggested an additional role for Nef in increasing viral production, possibly during the morphogenesis and release of new virions. To this end, first, Nef binds p6* in GagPol [27], which means that Nef travels with viral assembly intermediates inside cells and is incorporated into new viral particles. This association found strong genetic support when two different Nef proteins, one the naturally occurring allele of Nef (NefF12), the other engineered artificially from NefNL4-3 (NefKKXX), could retain GagPol near the ER and block subsequent processing and release of viral particles [27,28]. Second, Nef stimulates transcription from the viral LTR as well as of many cellular genes [58-60], which include those involved in cholesterol biosynthesis [61]. Indeed, Nef also binds cholesterol and can be found in DRMs [25], although one study disputes this localization [62]. In addition, like DRMs, internal vesicles of MVBs are enriched in cholesterol and harbor most of the cholesterol from the endocytic pathway [63]. Third, Nef binds PI3K, whose kinase activity is required for the formation of MVBs [42,64,65]. To this end, it is of interest that wortmannin, an inhibitor of PI3K, blocks the release of viral particles from cells [66]. Finally, why would the virus require a "modified" L domain, when ratios of Gag to GagPol are 20:1 in viral particles? Possibly, because GagPol is bulkier and/or otherwise contains additional retention signals in Pol, which represents one half of the polyprotein. Possibly, because Nef forms oligomers, it could increase the size of viral assembly intermediates that would be optimal for the targeting and egress of viral particles from the infected cell. Otherwise, Nef contains additional motifs that might be attractive to the virus at this stage of its replicative cycle. PI3K and lipids have been mentioned already, but Nef also associates with additional trafficking and signaling molecules. As both Nef and gp41 interact with AP complexes, some of these might facilitate the loading of Env onto viral particles [67]. Others cause cytoskeletal rearrangements and increase the local polymerization of actin, which is required not only for the formation of pseudopodia, from which virions bud, but also for the integrity of viral particles themselves [14,68]. In support of these findings, a recent study found that SIV Nef not only augments the incorporation of many retroviral glycoproteins onto Gag of SIV by increasing their co-localization in late endosomes but leads to greater egress of these pseudotyped viral particles from infected cells [31].

## Conclusion

From these studies emerges an additional effect of Nef on viral replication. During late stages of the viral replicative cycle, Nef behaves like a chaperone for HIV-1. By interacting with viral structural proteins and the ESCRT machinery, it facilitates the egress of optimally infectious progeny virions from infected mononuclear cells Future studies will evaluate the role of PI3K in this process as well as confirm these findings in the primate model of AIDS, with SIV in rhesus macaques.

## Methods

### Antibodies

Monoclonal α HA epitope (F7) (Santa Cruz Biotechnology, Santa Cruz, CA), monoclonal α V5 (Invitrogen, Carlsbad, CA), monoclonal α FlagM2 (Sigma-Aldrich, St. Louis, MO), monoclonal α Nef [25], and mouse α p24 (AG3.0) antibodies were used as first antibodies to detect epitope-tagged proteins, Nef and Gag, respectively. Secondary HRP-conjugated anti-mouse antibodies (Santa Cruz Biotechnology, Santa Cruz, CA) were detected by enhanced chemilumnescence (ECL, Amershan Biosciences, Evanston, IL). α AIP1 antibodies were a kind gift of Wesley Sundquist (U. of Utah, Salt Lake City, UT)

### Plasmid constructions

Plasmid DNAs encoding replication-competent HIV-1 proviruses were from HIV-1_NL4-3 _[69]. The *nef*-deleted variant NL4-3Δ Nef was generously provided by John Guatelli (U. of California, San Diego, CA). Proviral infectious clones for the macrophage-tropic viruses ADA and ELI, and the same clones disrupted for the Nef ORF (ADAΔ Nef, ELIΔ Nef) where provided by Marcelo Soares (Federal University, Rio de Janeiro, Brazil), and are described elsewhere [70,71]. Plasmid DNAs encoding *env*-deleted, *env *plus *nef*-deleted proviruses, and MLV-*env*, were kindly provided by Hirofumi Akari (NIH, Bethesda, MD) and are described elsewhere [72].

The Nef expression plasmid was generated by the amplification of the *nef *gene from the NL4-3 provirus and inserted into pcDNA3.1D (Invitrogen) at the TOPO site. This plasmid was used to derive the expression plasmids for the mutant NefΔ YPLF (Nef from NL4-3, residues deleted from positions 135 to 138), and the mutant NefYPL (Nef from NL4-3, mutated residues from positions 135 to 137 to alanines) proteins, by standard mutageneses. The human *Aip1 *cDNA was obtained from the American Type Culture Collection and was amplified by PCR with *Bam *HI (5') and EcoRI (3') restriction sites and inserted into pEF-BOS-HA (to obtain the HA epitope-tagged AIP1 protein) and into pGEX-4T1 (Pharmacia, Piscataway, NJ)(to obtain the GST.AIP1 fusion protein). pENX, which expresses Gag without p6, Env, Rev and Tat [33], was used to create pENX.Flag.Nef, which has a Flag eptiope-tagged Nef ORF at the C-terminus of the Gagp7 ORF. This plasmid expressed the mutant GagΔ p6.Nef chimera. pNL-Δ pol was derived from pNL-, which bears two mutations in the Gagp6 L domain (PTAP to LTAL). To generate the pNL-Δ pol plasmid, the entire *pol *gene together with the Vif and the Vpr ORFs were removed by *Bcl I*-*Sal I *digestion, treated with Klenow enzyme and further ligated with the T4 DNA ligase (both from Invitrogen). This plasmid expressed virus like particles (VLPs) that did not bud from cells. To generate the expression plasmid for the Myc.Vpr protein (pEF.Myc.Vpr), the *vpr *gene from HIV-1_NL4-3 _was inserted into pEF.BOS.Myc. For the expression of the hybrid Myc.Vpr.Nef protein, the *nef *gene from HIV-1_NL4-3 _was inserted into pEF.Myc.Vpr downstream from the *vpr *gene.

### Cells and transfections

293T and HeLa.CIITA cells were grown in DMEM with 10% FCS and antibiotics. Transfections were performed using Lipofectamine (Invitrogen). SupT1 cells were grown in RPMI1640 medium with 10% FCS, antibiotics and L-glutamine. Cells were electroporated using a BioRad electroporator (BioRad USA Life Sciences, Hercules, CA) as follows: 1 × 10^7 ^cells in the presence of 10 μg of DNA, electroporated at 200 V and 995 μF. Primary macrophage cultures were obtained from Peripheral Blood Mononuclear Cells (PBMCs) by their adherence to plastic. Briefly, PBMCs were obtained from buffy coats of anonymous, healthy blood donors and separated by centrifugation over Ficoll-Paque (Amershan Biosciences, Evanston, IL). 10^7 ^cells were incubated in DMEM with 5% human serum type A and antibiotics. PBMCs were left to sit on TC25 plastic bottles for 7 days. Transfections were performed using CaPO4 protocols (Stratagene, Carlsbad, CA). Transfected cells were analyzed 5 days later for production of viral partcles and intracellular levels of Nef.

### Virus and Gag VLP production, virion and Gag VLP isolation and Gag expression

To assess effects of Nef during the production of new viral particles, SupT1 cells were electroporated and macrophages were transfected with proviral DNAs and Nef expression plasmids at 1:1 molar ratios. 4 to 8 days later, cells and cell culture supernatants were harvested. The co-expression of mutant HIV-1_NL4-3_Δ Env or HIV-1_NL4-3_Δ EnvΔ Nef (which lacks the *nef *gene) plasmids with the MuLV Env at equivalent amounts generated pseudotyped viruses. For the evaluation of Gag VLPs, 293T cells were transfected with the pENX and the pENX.Flag.Nef proviral clones. 293T cells were also transfected with the pL- and pNL-Δ pol proviral clones together with the Vpr or Vpr.Nef fusion plasmids at different proportions of each plasmid, ranging from 1:1 to 1:5 of the pL- or pNL-Δ pol to the Vpr or hybrid Vpr.Nef plasmids. pENX and pL were kind gift of Paul Bieniasz (ADARC, NYC, NY) [36]. Culture supernatants were clarified at low-speed centrifugation, cleared through a 0.45 μm-pore-size filter (Millipore, Bedford, MA) and followed by ultracentrifugation through a 20% sucrose cushion at 100,000 × g for 1.5 h. Pellets were suspended in 1 × PBS overnight at 4°C. Viruses were lysed in SDS-loading buffer and viral protein contents were analyzed by western blotting. Quantification of virion production was performed by p24 capture ELISA (PerkinElmer/NEN Life Science Products, Boston, MA). Cells were lysed in radioimmunoprecipitation assay (RIPA) buffer (150 mM NaCl, 50 mM Tris [pH 7.2], 1% Triton X-100, 0.1% sodium dodecyl sulfate [SDS]), and viral protein content analyzed by western blotting. Cell-associated viral proteins were quantified as above.

### Protein purification, in vitro translation and GST pulldowns

The GST.AIP1 fusion protein was expressed in the BL21(DE3)pLysS strain of *E. coli *(Novagen, Madison, WI) and purified using Glutathione Sepharose beads (GE Healthcare Bio-Sciences AB, Uppsale, Sweden) with a modified lysis buffer (50 mM Hepes [pH 7.8], 100 mM KCl, 1% Triton X-100, 2 mM EDTA, 0.1 mM PMSF, and 1 μg/ml lysozyme). Coomassie blue staining of SDS-PAGE was used to check the purity of the GST.AIP1 chimera. Amounts of protein were determined by a protein assay kit (BioRad, Hercules, CA). Wild type and mutant Nef proteins were transcribed and translated using the rabbit reticulocyte in vitro (TNT, Promega, Madison, WI). SDS-PAGE and western blotting using αV5 antibodies was used to assess the quality of translated proteins. For *in vitro *binding assays, 0.5 μg of immobilized GST or hybrid GST.AIP1 proteins were incubated with 5 μl of V5 epitope-tagged proteins for 4 h at 4°C in 750 μl of CHAPS buffer (50 mM Tris-HCl [pH 7.4], 0.05 mM EDTA, 10 mM CHAPS and protease inhibitors). Beads were then washed 5 times in the same buffer and subjected to SDS-PAGE and western blotting.

### Co-Immunoprecipitation

293T cells were transfected with 0.5 μg of pCR.AIP1.HA [40] alone or co-transfected with 0.5 μg of plasmids expressing wild type or mutant NefYPL proteins. 36 h after the transfection, cells were harvested, washed, and disrupted by dounce homogenization in hypotonic buffer containing protease inhibitor cocktails (Sigma-Aldridge, Saint Louis, MI). After removing nuclei and unbroken cells, 5 μg/ml of α HA antibodies (Santa Cruz Biotech, Santa Cruz, CA) was added to the supernatant followed by proteinG-beads (GE Healthcare Bio-Sciences AB, Uppsale, Sweden). Immunoprecipitations were resolved by 12% SDS-PAGE, and Nef proteins were detected by western blotting using α Nef antibodies.

### Electron microscopy

HeLa.CIITA cells were transfected with peGFPN1 (Clontech Laboratories, Mountain View, CA) expressing GFP, Nef.GFP, or mutant NefYPL.GFP fusion proteins by Fugene6 (Roche Applied Science, Indianapolis, IN). 48 hours after the transfection, GFP-expressing cells were sorted by FacsVantage and fixed in a mixture of 3% glutaraldehyde and 1% paraformaldehyde, 0.1M cacodylate buffer, pH 7.4 prior to the process for ultra thin sectioning. 30 images of each sample were taken randomly, and the numbers of MVBs were quantified.

## Abbreviations

AIDS, acquired immunodeficiency syndrome; AIP1, apoptosis linked gene 2 (ALG2)-interacting protein 1; AP, adaptor protein complex; CA, capsid; Env, envelope; DRM, detergent resistant microdomains; EIAV, equine infectious anemia virus; ESCRT, endosomal sorting complex required for transport; Gag, group specific antigen; GagPol, Gag-polymerase; HIV, human immunodeficiency virus; L, late domain; MVB, multivesicular body; MIIC, major histocompatibility complex (MHC) class II compartment; Nef, negative factor; PI3K, phosphoinositide 3 kinase; PBMC, peripheral blood mononuclear cells; SIV, simian immunodeficiency virus; VLP, virus like particle; Tsg101, tumor suppressor gene 101.
